# Morphological and Redox/Glycative Alterations in the PCOS Oviducts: Modulating Effects of Carnitines in PCOS Mice

**DOI:** 10.3390/biology13120964

**Published:** 2024-11-23

**Authors:** Mariacarla De Rubeis, Ilaria Antenisca Mascitti, Domenica Cocciolone, Martina Placidi, Teresa Vergara, Giovanna Di Emidio, Guido Macchiarelli, Carla Tatone, Stefania Annarita Nottola, Maria Grazia Palmerini

**Affiliations:** 1 Department of Anatomy, Histology, Forensic Medicine and Orthopaedics, Sapienza University, 00161 Rome, Italy; mariacarla.derubeis@uniroma1.it (M.D.R.); stefania.nottola@uniroma1.it (S.A.N.); 2 Department of Life, Health and Environmental Sciences, University of L’Aquila, 67100 L’Aquila, Italy; ilariaantenisca.mascitti@graduate.univaq.it (I.A.M.); domenica.cocciolone@univaq.it (D.C.); martina.placidi@univaq.it (M.P.); teresa.vergara@guest.univaq.it (T.V.); giovanna.diemidio@univaq.it (G.D.E.); guido.macchiarelli@univaq.it (G.M.); carla.tatone@univaq.it (C.T.)

**Keywords:** PCOS, DHEA, oviducts, mouse, carnitines, histology, electron microscopy, glycative stress, oxidative stress

## Abstract

Polycystic ovarian syndrome (PCOS) is a heterogeneous condition characterized by hyperandrogenism (HA), polycystic ovaries, and dysfunctional ovulation, and it is associated with metabolic problems such as insulin resistance (IR) and obesity. After having investigated the morphological and antioxidant/antiglycative alterations on mouse ovaries and uteri, we here focus on PCOS oviducts, a tract of the reproductive system essential for the nourishment and transport of gametes and embryos. The modulating effects of L-carnitine (LC) and acetyl-L-carnitine (ALC) were also assessed. CD1 mice were administered or not with dehydroepiandrosterone (DHEA, 6 mg/100 g body weight) for 20 days, alone or with 0.40 mg of L-carnitine (LC) and 0.20 mg of acetyl-L-carnitine (ALC). Oviducts were then subjected to histology and immunohistochemistry to evaluate their morphology and collagen deposition, and steroidogenesis. Oxidative, mitochondrial, and methylglyoxal (MG)-dependent damage was also investigated. Transmission electron microscopy was used to detect ultrastructural alterations. The PCOS oviducts were affected by hyperfibrosis, hyperplasia, hypertrophy, and altered steroidogenesis, with oxidative alterations associated with MethylGlyoxal-Advanced Glycation End product (MG-AGE) accumulation. A reduced ciliary coverage and numerous dilated intercellular spaces were found in the epithelium. LC-ALC administration mitigated PCOS oviductal alterations. These results provide evidence for the detrimental action of oxidative and glycative stress in PCOS oviducts, confirming a protective role of carnitines on the PCOS phenotype.

## Simple Summary

Polycystic ovary syndrome (PCOS) is a multifactorial disorder associated with female infertility. We aimed to investigate the effects of L-carnitine and acetyl-L-carnitine. CD1 mice were administered or not with dehydroepiandrosterone, alone or with L-carnitine and acetyl-L-carnitine. Oviducts were subjected to histology and immunohistochemistry to evaluate their morphology, collagen deposition, and steroidogenesis. Oxidative, mitochondrial, and methylglyoxal-dependent damage was also investigated. Via transmission electron microscopy, we evaluated the ultrastructural alterations. PCOS oviducts were affected by hyperfibrosis, hyperplasia, hypertrophy, and altered steroidogenesis, with oxidative modifications; a reduced ciliary coverage and numerous dilated intercellular spaces were found in the epithelium. Oral supplementation of carnitines ameliorated the morphological alterations observed in PCOS oviducts, with an attenuation of oxidative and glycative stress.

## 1. Introduction

Polycystic ovarian syndrome (PCOS) is a heterogeneous condition characterized by hyperandrogenism (HA), polycystic ovaries, and dysfunctional ovulation, and it is associated with metabolic problems such as insulin resistance (IR) and obesity [1]. It is a reproductive and multifactorial disease affecting 8–13% of women of reproductive age [2], considered the most common cause of anovulatory infertility in women of reproductive age [3]. According to the Rotterdam criteria, the three main phenotype characteristics of this condition are hyperandrogenism, polycystic ovaries, and ovulatory dysfunction: two out of these three features (1. oligo and/or anovulation, 2. clinical and/or biochemical signs of hyperandrogenism, 3. polycystic ovaries and the exclusion of other aetiologies like congenital adrenal hyperplasias, androgen-secreting tumours, and Cushing’s syndrome) are required for a PCOS diagnosis (Rotterdam criteria) [4]. Women diagnosed with PCOS have various reproductive, metabolic, cardiovascular, and psychological comorbidities. A combination of genetic and environmental factors can predict the development of PCOS in women, type 2 diabetes mellitus (T2DM), IR, obstructive sleep apnea, and elevated blood pressure. PCOS may clinically manifest as HA, oligoanovulation, and polycystic ovary morphology (PCOM). IR appears to be a key factor in PCOS development, with the ovary being only one among the several organs affected. Nearly 44–70% of women diagnosed with PCOS have IR with endometrial IR and impaired glucose transport, leading to chronic low-grade inflammation, immune dysfunction modifications in the vascular uterus, elevated endometrial gene expression, and cellular abnormalities [1]. PCOS is associated also with cardiovascular problems, neurological and psychological effects (including anxiety and depression), and breast and endometrial cancers [5]. Environmental and genetic factors are known to play a role in the aetiology [6], but the molecular mechanisms leading to its establishment are still unclear, thus limiting the therapeutic strategies. Recently, we found in mice a central role for oxidative and glycative stresses in PCOS pathogenesis [7]. The role of natural antioxidants in promoting insulin sensitivity and modulating the inflammatory response seems to restore a functional balance in PCOS patients and animal models [8,9]. Moreover, diet supplementation with natural antioxidants leads to a significant improvement in the menstrual cyclicity, acne, and hirsutism in PCOS patients, with a significant body weight and BMI reduction [10]. In this regard, the role of carnitines has emerged from clinical and experimental research [11,12]. Carnitines play a key physiological role in lipid metabolism and intermediary metabolic pathways [13]. By shuttling acetyl groups from outside to inside the mitochondrial membrane, carnitines regulate energetic metabolism and sense the cellular energy levels [14]. For proper cell functioning, the whole carnitine pool needs to be present, with L-carnitine (LC) representing 50–85% of the pool and acetyl-L-carnitine (ALC) being the most abundant form of acyl-carnitines. Given the different effects of LC and ALC on cellular respiration, the combined presence of LC and ALC has been known to support the metabolic flexibility [15]. The ALC/LC ratio ranging from 0.3 to 0.5 is tissue-specific, and its changes are evidence of a disturbed mitochondrial metabolism [14]. LC is essential in fatty acid metabolism by facilitating the transport of long-chain free fatty acids into the mitochondrial matrix, making them available for beta-oxidation. It is also involved in alpha-ketoacids’ intra-mitochondrial shuttling and oxidation and modulates the intramitochondrial acyl-CoA/CoA ratio. Moreover, LC shuttles acetyl groups from inside to outside the mitochondrial membrane, thus regulating glucose metabolism and sensing the cellular energy levels. LC has direct antioxidant properties, protects the mitochondrial metabolism, and modulates the activities of ROS-producing enzymes. For these reasons, it may contribute to the treatment of PCOS and its complications linked to redox imbalance [15]. In a recent work from our research group, carnitine formulations containing LC and acetyl-L-carnitine (ALC) orally administered to a DHEA-induced PCOS mouse model were effective in ameliorating ovarian function [16].

This is in agreement with the presence of low seric levels of carnitines in obese and non-obese PCOS women, in association with insulin resistance and hyperandrogenism [17,18,19,20]. Also, others found that LC administration improved the menstrual cycle regularity, ovulation, and pregnancy rates [19].

To further investigate the potential role of carnitine supplementation in the female reproductive system, we here focus our attention on the oviducts, the site of gamete transport, fertilization, and embryogenesis [21]. It is known that the excess of testosterone involved in PCOS pathogenesis can lead to tubal dysfunctions such as blockages via mucus accumulation in the oviducts and via epithelial breakdown and luminal collapse in the isthmus, with evident consequences for fertility along with negative effects on cilia beating [22,23]. However, studies regarding PCOS-related changes in the oviducts are scarce in animal models. In the mouse, the oviduct is a coiled, complex structure consisting of four segments: the infundibulum, ampulla, isthmus, and uterotubal junction [24]. In this study, we investigated whether PCOS is associated with the morphofunctional alterations of murine oviducts in a DHEA-induced model of PCOS and evaluated if the administration of LC-ALC could ameliorate the characteristic PCOS-induced changes in the ampullar portion of the oviducts. For these purposes, morphological analysis by using light and transmission electron microscopy was associated with searching for molecular alterations as evidence of oxidative/glycative damage, fibrosis, and mitochondrial and steroidogenic dysfunctions.

## 2. Materials and Methods

### 2.1. Animals

Outbred CD-1 mice (Charles River Italia s.r.l., Calco, Italy) were maintained in a temperature-controlled environment under a 12 h light/dark cycle (7.00–19.00) and free access to feed and water ad libitum. All the experiments were carried out in conformity with national and international laws and policies. The project was approved by the Italian Ministry of Health and the internal Committee of the University of L’Aquila (Authorization n. 269/2018-PR).

Four-week-old CD-1 female mice, with a body weight of 20–21 g, were randomly assigned to four groups (10 mice per group). The PCOS model was established as previously described [7]. Briefly, the mice were daily sub-cutaneously injected with DHEA (6 mg/100 g body weight, 100 μL/mouse in sesame oil with 10% of 95% ethanol, Sigma) for 20 consecutive days (DHEA group). The vehicle control group was injected with 0.09 mL of sesame oil and 0.01 mL of 95% ethanol daily for 20 consecutive days (control group). At the same time, the mice received, via oral gavage, a carnitine formulation made with 0.40 mg of L-carnitine and 0.20 mg of acetyl-L-carnitine (DHEA/LC-ALC group) dissolved in water, 100 µL/mouse daily for 20 consecutive days. The control and DHEA groups received daily oral administration of water for 20 consecutive days. The mice were sacrificed by administering an inhalant overdose of carbon dioxide (CO_2_, 10–30%), followed by cervical dislocation. All efforts were made to minimize suffering. After having opened the peritoneal cavity, the oviducts were removed from each side and washed in phosphate-buffered saline (PBS) solution before further processing.

### 2.2. H&E Staining and Morphometric Analysis

After being washed in PBS, the right oviducts were fixed in 3.7% paraformaldehyde (PFA) in PBS (Bio-Optica, Milan, Italy) for 12–16 h, carefully washed in PBS, dehydrated in the ascending series of alcohol, clarified in xylene, and embedded in paraffin blocks. Sections were cut using a microtome (Leica SMR2000, Wetzlar, Germany) and sliced into 6 µm serial sections. The sections were then deparaffined and hydrated through xylenes and a descending series of alcohol, stained with H&E according to the manufacturer’s instruction (Bio Optica, Milan, Italy), and observed via light microscopy (Zeiss Axiostar Plus, Oberkochen, Germany). The luminal epithelial cell height was measured from the apical surface to the basement membrane, and oviductal thickness (height) measurements were investigated according to Kuyuku and colleagues, using the ImageJ software (https://imagej.net/, accessed on 7 November 2024). All the experiments were repeated at least three times, and the data are expressed as mean ± S.D.

### 2.3. Mallory Trichrome Staining

The paraffin-embedded sections of the formalin-fixed oviducts were deparaffinized and hydrated through xylenes and graduated alcohol series and processed for Mallory Trichome staining (Bio Optica, Milan, Italy), according to the manufacturer’s instructions. The sections were observed via light microscopy (Zeiss Axiostar Plus, Oberkochen, Germany).

### 2.4. Immunohistochemical and Morphometric Analysis: MG-AGE and 4-HNE

The paraffin-embedded sections of the formalin-fixed oviducts were deparaffinized and hydrated through xylenes and graded alcohol series. To increase the immunoreactivity, the sections were boiled in 10 mM citrate buffer (pH: 6.1. Bio-Optica, Milan, Italy) in a microwave at 720 W (3 cycles/3 min each). Then, the sections were subjected to treatment for blocking endogenous peroxidase activity (Dako). After being thoroughly washed, the sections were incubated with mouse on mouse (M.O.M.) IgG blocking reagent overnight at 4 °C (Vector Laboratories) according to the manufacturer’s protocol. The sections were, then, incubated with mouse monoclonal to methylglyoxal (MG)-AGE (Arg-Pyridine, AGE06B, BioLogo, 1:100) antibody or rabbit polyclonal to 4-HNE (4-Hydroxynonenal, ab46545, Abcam, 1:100) diluted in M.O.M. diluent for 40 min, according to the Vector Laboratories’ instructions. MG-AGE and 4-HNE were revealed by using Labelled Polymer-HRP, 3,3-diaminobenzidine (DAB) substrate buffer, and DAB (DAKO kit), according to the manufacturer’s instructions. Counterstaining was performed using hematoxylin (Bio-Optica). A negative control was performed by omitting the primary antibody and substituting it with M.O.M. diluent alone. The sections were dehydrated, mounted with Neomount (Merck, Darmstadt, Germany), observed, and photographed under a Leiz Laborlux S microscope (Oberkochen, Germany) equipped with an Olympus digital compact camera. The evaluation and automated scoring of immunohistochemistry (IHC) signals were performed by using the Image J 1.44p software (IHC profiler plugin) [7]. All the data are presented as mean ± SEM of the mean pixel intensity. The statistical analysis was assessed via ANOVA followed by Tukey’s HSD test. The analyses were performed using the SigmaStat software 4.0 (Jandel Scientific Corporation, San Rafael, CA, USA). A *p*-value < 0.05 was considered statistically significant.

### 2.5. Immunohistochemical Analysis and Morphometric Analysis: TOMM20, Col1, and 17 β-HSD IV

The paraffin-embedded sections of the formalin-fixed oviducts were deparaffinized and hydrated through xylenes and graded alcohol series. To increase the immunoreactivity, the sections were boiled in 10 mM citrate buffer (pH 6.1, Bio-Optica, Milan, Italy) in a microwave at 720 W (3 cycles/3 min each). Then, the sections were subjected to treatment for blocking the endogenous peroxidase activity (Dako). After being thoroughly washed, the sections were incubated with 5% BSA/PBS (Sigma-Aldrich, St. Louis, MO, USA) for 1 h at RT. Then, the oviduct sections were incubated with the following primary antibodies: rabbit polyclonal to 17 β-HSD IV, TOMM20 (1:100 and 1:400, respectively) (Thermo Fisher Scientific, Rockford, IL, USA) and rabbit polyclonal to COL1 (1:500) (Immunological Sciences Ab-82138, Rome, Italy), all diluted in 1% BSA/PBS overnight at 4 °C. After being washed with PBS, the rabbit antibodies were revealed by using Labelled Polymer-HRP, DAB substrate buffer, and DAB (DAKO kit, Glostrup, Denmark), according to the manufacturer’s instructions. Counterstaining was performed with hematoxylin (Bio-Optica). A negative control was performed by omitting the primary antibody and substituting it with 1% BSA/PBS diluent alone. The sections were dehydrated and mounted with Neomount (Merck, Darmstadt, Germany). They were observed and photographed under a Leitz Laborlux S microscope (Oberkochen, Germany) equipped with an Olympus digital compact camera. The evaluation and automated scoring of IHC signals were performed by using the same methos as described in Section 2.4.

### 2.6. Light Microscopy (LM) and Transmission Electron Microscopy (TEM)

The remaining oviducts from each experimental group were isolated from their neighbouring ovaries, gently washed in PBS solution, cut into small pieces and immediately fixed in 2.5% glutaraldehyde (Agar Scientific, Stansted, UK)/0.1 M PBS. Fixed samples were maintained at 4 °C for at least 48 h until the next preparative for TEM; successively, the oviductal pieces were rinsed in PBS, post-fixed with 1% osmium tetroxide (Agar Scientific)/0.1 M PBS, and rinsed again in 0.1 M PBS. The samples were then dehydrated in ascending series of ethanol (Carlo Erba Reagenti, Milan, Italy), immersed in propylene oxide (BDH Italia, Milan, Italy) for solvent substitution, and embedded in epoxy resin EMbed-812 (Electron Microscopy Sciences, Hatfield, PA, USA). Semithin sections (1 mm thick) were stained with Methylene Blue, examined using a LM (Zeiss Axioskop, Oberkochen, Germany), and photographed using a digital camera (DFC230; Leica, Wetzlar, Germany). Ultrathin sections (60–80 nm) were cut using a diamond knife on a Reichert-Jung Ultracut E ultramicrotome (Reichert Technologies, Munich, Germany), mounted on copper grids, and contrasted with Uranyless (Electron Microscopy Sciences, Hatfield, PA, USA) followed by lead citrate (SIC, Rome, Italy). They were examined and photographed using Zeiss EM10 and Philips TEM CM100 Electron Microscopes operating at 80 kV.

## 3. Results

### 3.1. Oviductal Hyperplasia Hypertrophy and Fibrosis

**Figure 1:**
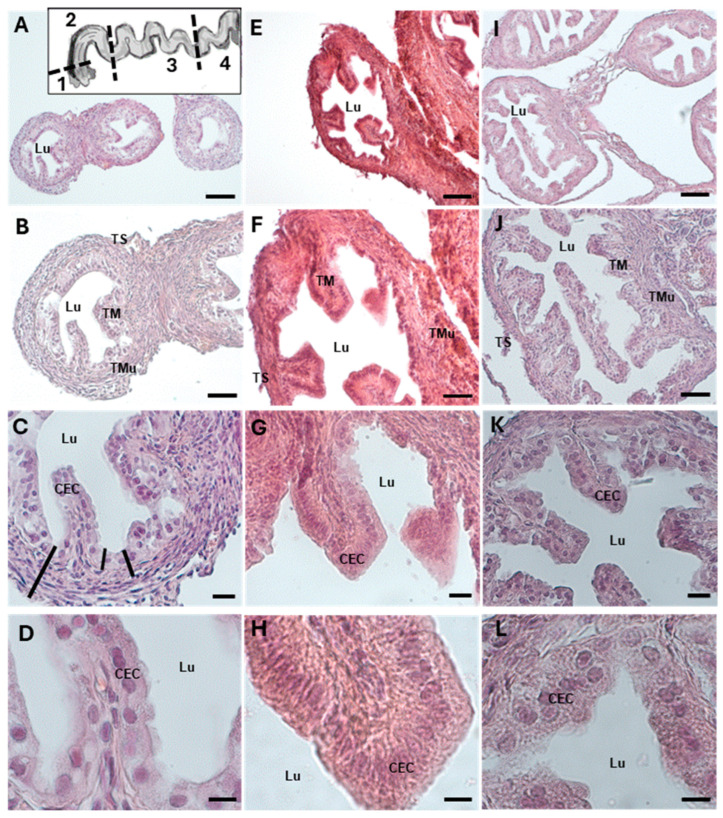
HE staining of mouse ampullar oviducts in control (**A**–**D**), DHEA (**E**–**H**), DHEA/LC-ALC (**I**–**L**) groups. (**A**,**E**,**I**): low-magnification LM pictures of the lumen (Lu). Inset in (**A**) indicates the four segments of the oviducts to evidence the ampullar portion (1: infundibulum; 2: ampulla; 3: isthmus; 4: intramural portion). (**B**,**F**,**J**): tunica mucosa (TM), tunica muscolaris (TMu), and tunica serosa (TS). (**C**,**D**,**G**,**H**,**K**,**L**): high magnification of the luminal epithelium made of ciliated columnar cells (CEC). (**A**,**E**,**I**): LM, mag. 10×; bar: 100 µm. (**B**,**F**,**J**): LM, mag. 20×; bar: 50 µm. (**C**,**G**,**K**): LM, mag. 40×; bar: 20 µm. (**D**,**H**,**L**): LM, mag. 100×, bar 10 µm. Black bars in (**C**) label the thickness of the structures compared in Table 1 (from left to right: ampullary wall, epithelium, and mucosa).

In all the groups, the histological investigations evidenced a well-preserved microscopic organization of the murine ampullary oviducts. In the control group, the HE staining on the transversal sections underlined the typical organization in three layers: the inner tunica mucosa, the intermedia tunica muscularis, and the outer tunica serosa (Figure 1). The tunica mucosa was characterized by an epithelium of columnar cells, with numerous cilia, and an underlying lamina propria. The mucosa layer was richly folded (Figure 1A–D). The mean width of the oviductal epithelium, tunica mucosa, and ampullary wall was, respectively, 19.132 ± 5.982 μm, 28.943 ± 5.323 μm, and 63.771 ± 8.608 μm (Table 1).

In the DHEA group, the epithelial thickness increased due to the evident hyperplasia and hypertrophy of the epithelial cells, more proliferative in this group with respect to the controls (Figure 1E–H). The mean width of the oviductal epithelium, tunica mucosa, and ampullary wall in the DHEA group was significantly higher than in the controls, being, respectively, 36.971 ± 5.565 μm, 42.323 ± 1.535 μm, and 86.393 ± 11.209 μm (Table 1).

In the DHEA/LC-ALC group, hypertrophy and hyperplasia were reduced, but not significantly, if compared with the DHEA group, showing an aspect more similar to the controls (Figure 1I–L). The mean width of the epithelium, tunica mucosa, and ampullary wall in the DHEA/LC-ALC group was, respectively, 22.585 ± 7.814 μm, 34.399 ± 6.628 μm, and 73.117 ± 8.949 μm (Table 1).

**Figure 2:**
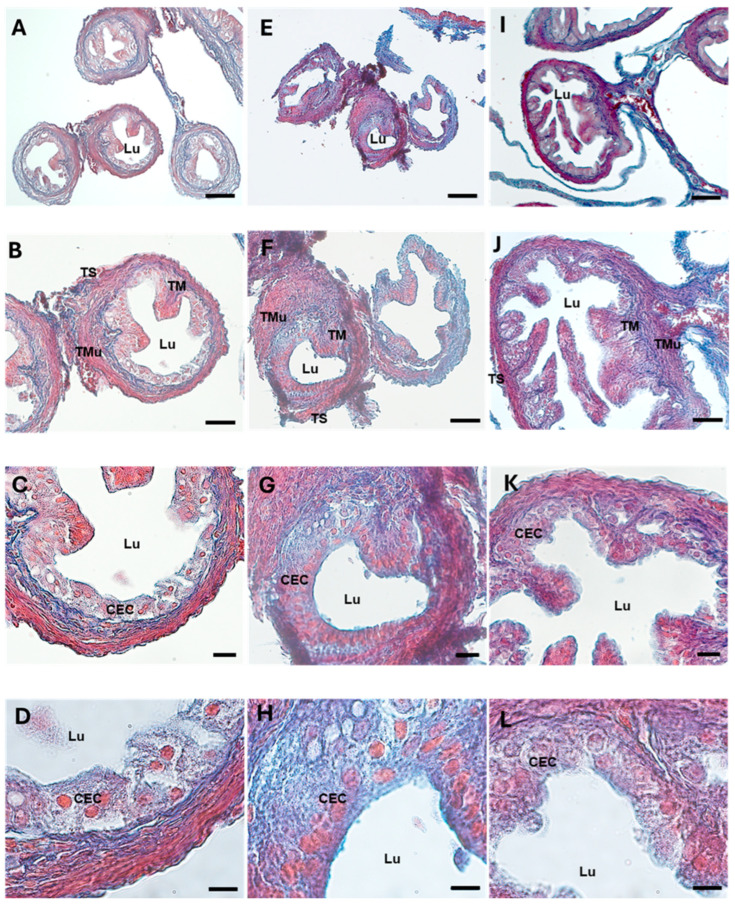
Mallory Trichrome staining of mouse ampullar oviducts in control (**A**–**D**), DHEA (**E**–**H**), DHEA/LC-ALC (**I**–**L**) groups. (**A**,**E**,**I**): low-magnification LM pictures of the lumen (Lu). (**B**,**F**,**J**): tunica mucosa (TM), tunica muscolaris (TMu), and tunica serosa (TS). (**C**,**D**,**G**,**H**,**K**,**L**): high magnification of the luminal epithelium made of ciliated columnar cells (CEC). (**A**,**E**,**I**): LM, mag. 10×; bar: 100 µm. (**B**,**F**,**J**): LM, mag. 20×; bar: 50 µm. (**C**,**G**,**K**): LM, mag. 40×; bar: 20 µm. (**D**,**H**,**L**): mag. 100×; bar 10 µm.

**Figure 3:**
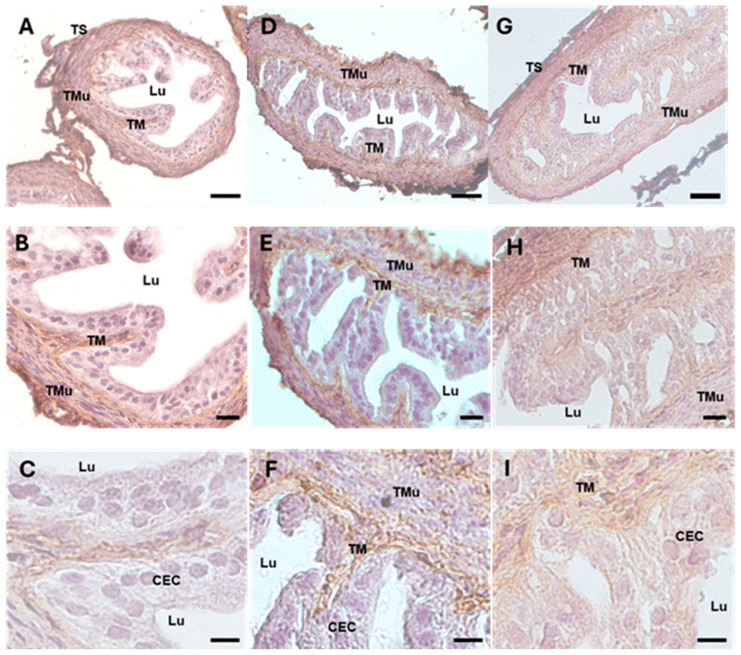
Col1 immunostaining of mouse ampullar oviducts in control (**A**–**C**), DHEA (**D**–**F**), and DHEA/LC-ALC (**G**–**I**) groups. (**A**,**B**,**D**,**E**,**G**,**H**): low-magnification LM pictures of the lumen (Lu), tunica mucosa (TM), tunica muscolaris (TMu), and tunica serosa (TS). (**C**,**F**,**I**): high magnification of the luminal epithelium made of ciliated columnar cells (CEC). (**A**,**D**,**G**): LM, mag. 20×; bar: 50 µm. (**B**,**E**,**H**): LM, mag. 40×; bar: 20 µm. (**C**,**F**,**I**): LM, mag. 100×; bar: 10 µm.

A high level of fibrosis was observed via Azan Mallory and type 1 collagen (Col1) staining in the DHEA group (Figure 2E–H and Figure 3D–F), where the collagen deposition was present not only in the tunica mucosa, as in the controls, but also in the muscular layer. In the DHEA/LC-ALC group (Figure 2I–L and Figure 3G–I), the fibrotic aspect resulted in being attenuated, if compared to the DHEA group, with weak staining, more similar to the controls. The morphometric analysis showed a significant increase in Col1 in the DHEA group, compared to the controls (respectively, 114.198 ± 14.431 vs. 151.102 ± 6.069, *p* < 0.05; Table 2), with a slight decrease in the DHEA/LC-ALC group (134.757 ± 12.856, *p* > 0.05) (Table 2).

### 3.2. Steroidogenesis (17 β-HSD IV)

**Figure 4:**
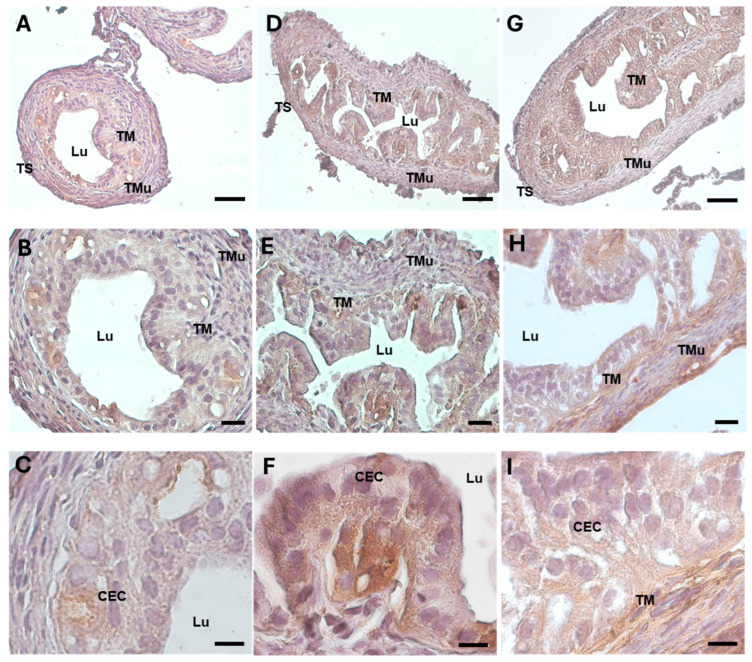
17 β-HSD IV immunostaining of mouse ampullar oviducts in control (**A**–**C**), DHEA (**D**–**F**), and DHEA/LC-ALC (**G**–**I**) groups. (**A**,**B**,**D**,**E**,**G**,**H**): low-magnification LM pictures of the lumen (Lu), tunica mucosa (TM), tunica muscolaris (TMu), and tunica serosa (TS). (**C**,**F**,**I**): high magnification of the luminal epithelium made of ciliated columnar cells (CEC). (**A**,**D**,**G**): LM, mag. 20×; bar: 50 µm. (**B**,**E**,**H**): LM, mag. 40×; bar: 20 µm. (**C**,**F**,**I**): LM, mag. 100×; bar: 10 µm.

The immunohistochemical and morphometric analysis of 17β-HSD IV, the steroidogenic enzyme that inactivates estradiol, revealed a significantly increased expression in the ampullar mucosa of the DHEA group (Figure 4D–F) (159.320 ± 10.313), if compared to the controls (Figure 4A–C) (112.175 ± 18.9, *p* < 0.05; Table 2). In the DHEA/LC-ALC group, the expression of 17 β-HSD IV was still evident, mainly in the lamina propria (Figure 4G–I), with a non-significant reduction compared to the DHEA group (140.502 ± 16.471, *p* < 0.05) (Table 2).

### 3.3. Oxidative Damage (4-HNE)

**Figure 5:**
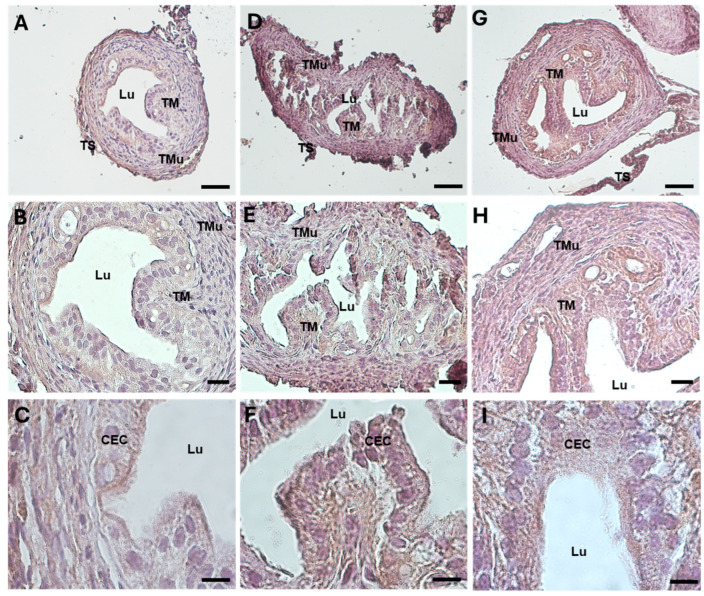
4-HNE immunostaining of mouse ampullar oviducts in control (**A**–**C**), DHEA (**D**–**F**), and DHEA/LC-ALC (**G**–**I**) groups. (**A**,**B**,**D**,**E**,**G**,**H**): low-magnification LM pictures of the lumen (Lu), tunica mucosa (TM), tunica muscolaris (TMu), and tunica serosa (TS). (**C**,**F**,**I**): high magnification of the luminal epithelium made of ciliated columnar cells. (**A**,**D**,**G**): LM, mag. 20×; bar: 50 µm. (**B**,**E**,**H**): mag. 40×; bar: 20 µm. (**C**,**F**,**I**): mag. 100×; bar: 10 µm.

The oxidative damage in the oviducts was evaluated by examining the expression pattern of the 4-hydroxynonenal (HNE) protein, a marker of lipid peroxidation and oxidative stress. While in the control group, the 4-HNE was weakly detected (Figure 5A–C) (87.178 ± 6.318, Table 2), the protein was significantly represented in the ampullar mucosa of the DHEA group (Figure 5D–F) (151.149 ± 1.590, *p* < 0.001) (Table 2). The marker showed a mild, non-significant decrease in the DHEA/LC-ALC group with respect to the DHEA group (Figure 5G–I) (148.670 ± 5.831, *p* < 0.05) (Table 2).

### 3.4. Mitochondrial Damage (Tomm20)

**Figure 6:**
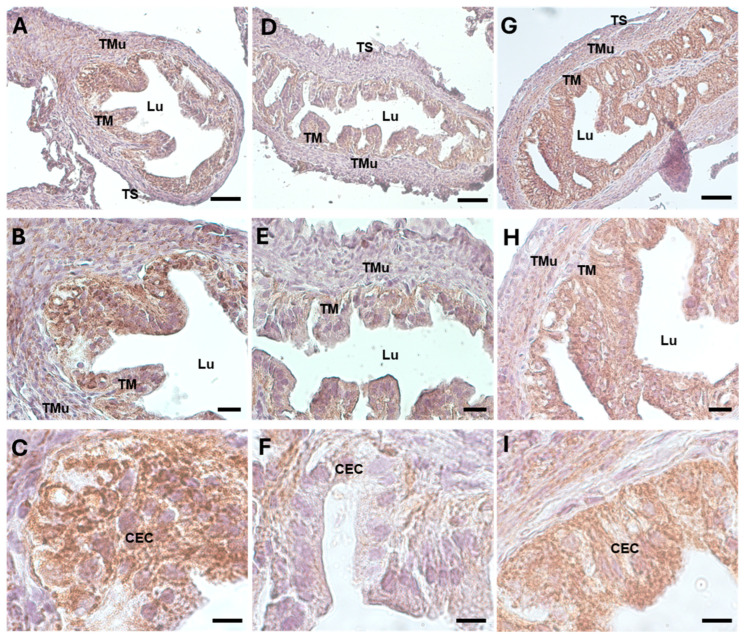
Tomm20 immunostaining of mouse ampullar oviducts in control (**A**–**C**), DHEA (**D**–**F**), and DHEA/LC-ALC (**G**–**I**) groups. (**A**,**B**,**D**,**E**,**G**,**H**): low-magnification LM pictures of the lumen (Lu), tunica mucosa (TM), tunica muscolaris (TMu), and tunica serosa (TS). (**C**,**F**,**I**): high magnification of the luminal epithelium made of ciliated columnar cells. (**A**,**D**,**G**): LM, mag. 20×; bar: 50 µm. (**B**,**E**,**H**): LM, mag. 40×; bar: 20 µm. (**C**,**F**,**I**): LM, mag. 100×, bar: 10 µm.

The oviductal mitochondrial activity was investigated by using Tomm20 immunostaining, a translocase of the outer mitochondrial membrane. Tomm20 was highly visible in the ampulla of the control group, especially in the tunica mucosa (Figure 6A–C) (163.698 ± 2.331, Table 2). Differently, its immunostaining was significantly reduced in the DHEA group (Figure 6D–F) (128.366 ± 3.474, *p* < 0.01). After carnitine treatment, Tomm20 significantly increased to levels more comparable to those in the control group (Figure 6G–I) (155.314 ± 9.732, *p* < 0.05).

### 3.5. Glycative Damage (MG-AGE)

**Figure 7:**
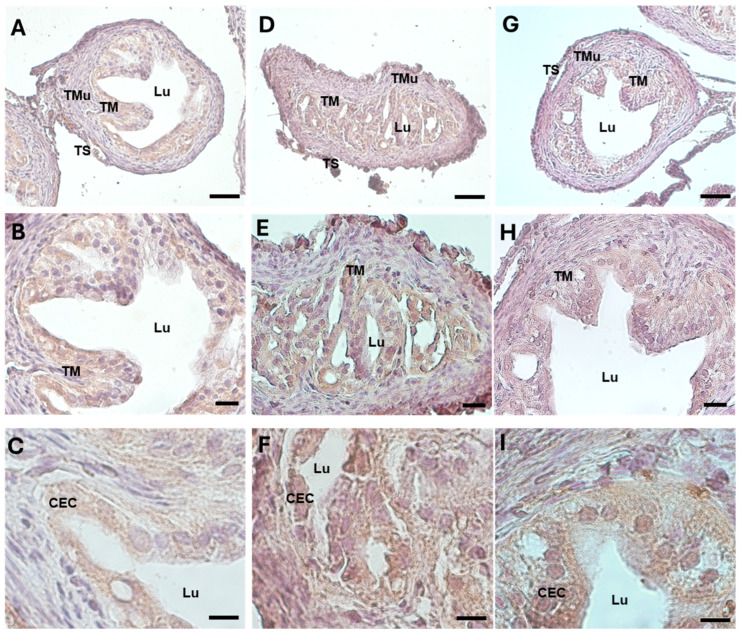
MG-AGE immunostaining of mouse ampullar oviducts in control (**A**–**C**), DHEA (**D**–**F**), and DHEA/LC-ALC (**G**–**I**) groups. (**A**,**B**,**D**,**E**,**G**,**H**): low-magnification LM pictures of the lumen (Lu), tunica mucosa (TM), tunica muscolaris (TMu), and tunica serosa (TS). (**C**,**F**,**I**): high magnification of the luminal epithelium made of ciliated columnar cells. (**A**,**D**,**G**): LM, mag. 20×; bar: 50 µm. (**B**,**E**,**H**): LM, mag. 40×; bar: 20 µm. (**C**,**F**,**I**): LM, mag. 100×, bar: 10 µm.

MG-AGEs strongly accumulated in the whole mucosa layer in the DHEA group (Figure 7D–F) with respect to the controls, where it was mainly located in the epithelial cells (Figure 7A–C) (respectively, 140.427 ± 5.868 vs. 126.053 ± 12.862, *p* < 0.05) (Table 2). A reduction in the glycative stress levels was detected in the DHEA/LC-ALC group, as evidenced by the less intense staining (Figure 7G–I) (133.279 ± 15.217) (Table 2).

### 3.6. TEM Analysis

#### 3.6.1. Control Group

**Figure 8:**
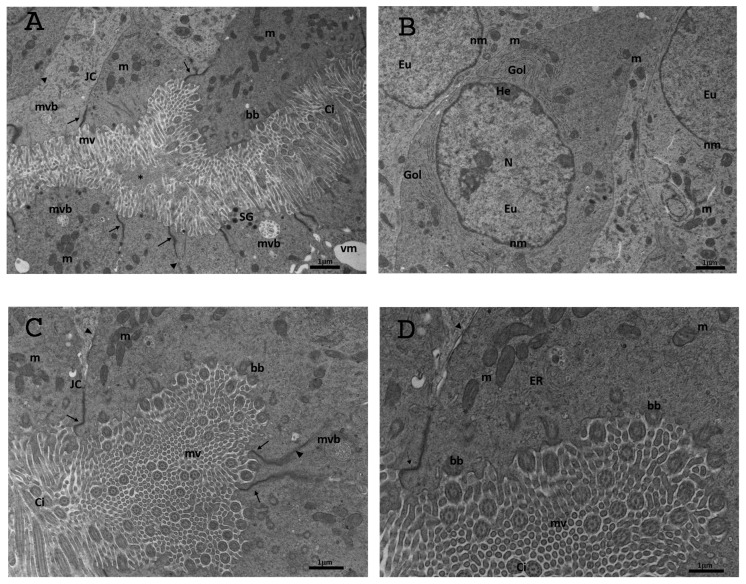
Representative TEM micrographs of the control group. (**A**) Ultrastructure of the columnar epithelium of TM, showing cilia (Ci) and microvilli (mv) extended from the luminal edge of the cell. The cytoplasm contains numerous elongated and oval-shaped mitochondria (m) with lamellar cristae; electron-dense secretory granules (SGs) and junctional complexes (JC) connected by apical zonulae occludens (arrow), followed by zonulae adhaerens (arrowhead); multivesicular bodies (mvb); granular filamentous secretion (asterisks). Bar: 1 mm; (**B**) CECs of TM showing a well-developed Golgi apparatus (Gol) and well-defined nuclei (N). Heterochromatin (He) was clustered in clumps or located as marginal patches under the nuclear envelope among the dispersed euchromatin (Eu). Bar: 1 μm; (**C**) the surface of TM shows numerous motile cilia (Ci) and short or long microvilli (mv) protruding into the lumen. Junctional complexes between neighbouring epithelial cells were well developed; basal body (bb). Bar: 1 μm; (**D**) high magnification of the surface of TM. ER: endoplasmic reticulum. Bar: 1 μm.

The epithelium of the oviductal ampullae via TEM analysis was simple columnar and consisted of ciliated and non-ciliated secretory cells. Cilia and microvilli extended from the luminal edge of the cell. The microvilli at the apical surface of the non-ciliated cells were short and stubby, while those of the ciliated cells were long and thin (Figure 8A). Motile cilia, protruding into the lumen, consisted of an evident axoneme of nine peripheral doublet microtubules, surrounding a central complex with two central microtubules and the central sheath (9 + 2 arrangement) (Figure 8A–D). Throughout the ampullar lumen, a granular filamentous secretion, produced by non-ciliated secretory cells, was present; the secretion occupied the spaces between the cilia of ciliated cells and was intimately associated with the cilia. The mechanism of secretion appeared to be apocrine (Figure 8A). The cytoplasm contained many elongated and oval-shaped mitochondria with lamellar cristae; a developed rough endoplasmic reticulum and Golgi complexes with a large number of vesicles were visible (Figure 8D). Aggregations of small multivesicular bodies and few vesicles appeared in the cytoplasm. Numerous electron-dense secretory granules were noticeable in the apical cytoplasm (Figure 8A). The nuclei, with irregular contours, were situated in the basal portion of the non-ciliated cells. Inside the nucleus, the chromatin appeared abundant and uniformly distributed, occasionally with heterochromatin clustered in clumps or located as marginal patches under the nuclear membrane (Figure 8B). Junctional complexes between neighbouring epithelial cells were well developed, and adjacent epithelial cells were connected by apical zonulae occludens, followed by zonulae adhaerens (Figure 8A,C,D).

#### 3.6.2. DHEA

**Figure 9:**
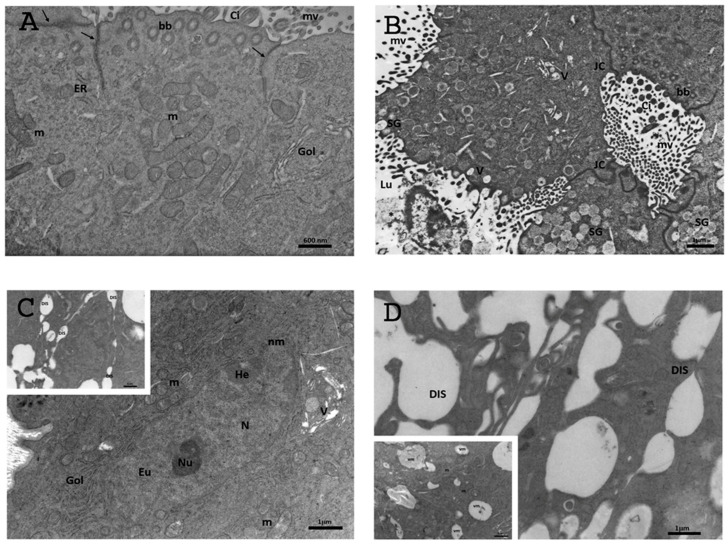
Representative TEM micrographs of the DHEA group. (**A**) Ultrastructure of the columnar epithelium of TM, showing numerous round or elongated mitochondria (m), a well-developed Golgi apparatus (Gol) and tubular elements of the endoplasmic reticulum (ER); motile cilia (Ci); multivesicular bodies (mvb); zonulae occludens (arrow); basal body (bb). Bar: 600 nm; (**B**) secretory cells contained many secretory granules (SGs) located at the apex of the cells. The lumen (Lu) contains membrane fragments and secretion droplets (asterisks); small vesicles (V); microvilli (mv); junctional complexes (JC); basal body (bb); cilia (Ci). Bar: 1 mm; (**C**) magnified micrograph of CECs with an evident nucleus (N) containing patches of heterochromatin (He) clustered under the nuclear membrane (nm) surrounded by mitochondria (m) and vesicles (V). Bar: 1 mm; Inset. The tunica mucosa is characterized by dilated intercellular spaces (DISs). Bar: 1 mm; (**D**) detail of dilated intercellular spaces (DISs). Bar: 1 mm; Inset. Mitophagic vacuoles (vm). Bar: 1 mm.

The ultrastructural analysis of the oviduct in the DHEA group showed a tunica mucosa with a simple columnar epithelium, with ciliated and non-ciliated cells (Figure 9A). The cilia were less abundant than in the control group, although the basal bodies were observed migrating to the apical surface, where cilia formation begins (Figure 9A,B). The mitochondria were elongated and oval shaped and were scattered throughout the cytoplasm (Figure 9A,C). The rough endoplasmic reticulum (RER) and Golgi apparatus were well developed. Short, stubby, irregularly shaped microvilli projected from the apical surface of each cell (Figure 9B). Secretory cells contained many secretory granules aggregated with each other; they were located at the apex of the cells and contained very small particles with an electron-lucent matrix (Figure 9B). The ampullary lumen in the DHEA group contained less granular-filamentous secretion than in the controls, except for a few membrane fragments and occasional segregated secretion droplets (Figure 9B). Roundish-to-amoeboid-shaped nuclei were located in the basal portion of the cells and showed an abundant organization of electron-dense heterochromatin, with dense nucleoli and a less electron-dense nuclear membrane than those observed in the control group (Figure 9C). The tunica mucosa showed well-preserved and organized intercellular junctions, as in the controls (Figure 9A,B); however, dilated intercellular spaces (DISs) were observed, confirming the presence of evident hyperplasia and hypertrophy, as observed in the histological investigations (Figure 9C,D, inset). Small endocytotic vesicles were observed in the apical cytoplasm (Figure 9B). Numerous mitophagic vacuoles and vesicles were observed (Figure 2B,D, inset to Figure 2D).

#### 3.6.3. DHEA/LC-ALC

**Figure 10:**
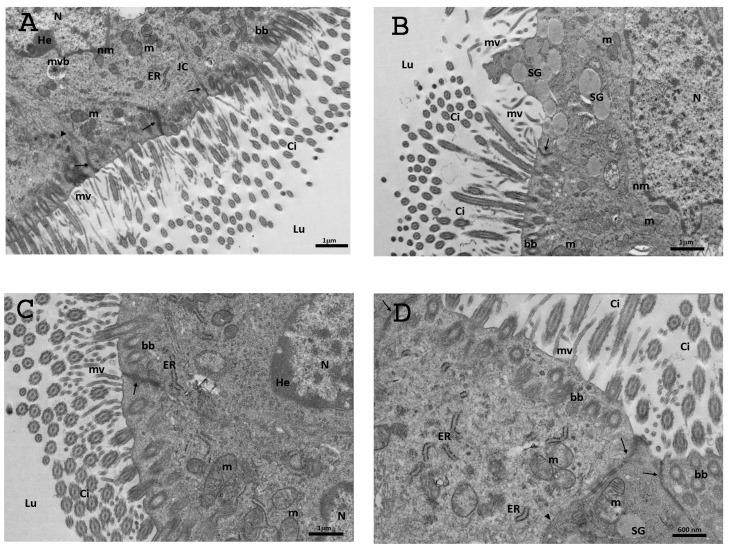
Representative TEM micrographs of the DHEA/LC-ALC group. (**A**) Micrograph of the tunica mucosa showing ciliated cells with an abundant population of long and well-preserved cilia (Ci), interspersed between microvilli (mv). Inside the cytoplasm well-preserved mitochondria (m), endoplasmic reticulum (ER) and Golgi apparatus (Gol) are visible. The nuclei are visible with abundant euchromatin (Eu) and heterochromatin (He), clustered in clumps or located as marginal patches under the nuclear membrane (nm); junctional complexes (JC); multivesicular bodies (mvb); basal body (bb) Bar: 1 mm; (**B**) epithelial cells showing the location of secretory granules (SGs), typically restricted to the apical cytoplasm. Inside the cytoplasm an evident nucleus with abundant euchromatin (Eu) uniformly distributed with heterochromatin (He) clustered in clumps; mitochondria (m); basal body (bb); zonulae occludens (arrow). Bar: 1 mm; (**C**) the surface of tunica mucosa showing a greater number of well-preserved mitochondria (m) with electron-dense lamellar cristae, endoplasmic reticular cisterna (ER), and evident nuclei (N); cilia (Ci); microvilli (mv); lumen (Lu); basal body (bb). Bar: 3 mm; (**D**) magnified micrograph of CECs showing numerous cilia (Ci), microvilli (mv), mitochondria (m) endoplasmic reticulum (ER), basal body (bb), zonulae occludens (arrow), and zonulae adhaerens (arrowhead). Bar: 600 nm.

After the administration of LC-ALC, the TEM examination of the ampullary epithelium of oviducts showed the presence of an abundant population of ciliated cells. The ciliated cells possessed long and well-preserved cilia, interspersed between the microvilli at their apical surface (Figure 10A–D). The cross-sections of the cilia are well visible in Figure 3C,D, which shows the nine pairs of peripheral doublet microtubules, surrounding a central complex with two central microtubules. The basal bodies forming the cilia were detected (Figure 10B–D). The luminal side of the mucosa also showed numerous long microvilli. Inside the cytoplasm, there was a greater number of well-preserved mitochondria, particularly in the supranuclear zone, with electron-dense lamellar cristae (Figure 10A–D). The endoplasmic reticular cisterna and the Golgi apparatus were present (Figure 10A,C,D). The intracellular location of granules was typically restricted to the supranuclear and apical cytoplasm (Figure 10B) although a few isolated granules could be observed elsewhere in the cytoplasm, with their electron-lucent content. Any secretory activity was observed. The nuclei with irregular contours were occasionally situated in the apical portion of the ciliated cells; they were delimited by a continuous and electron-dense nuclear membrane. The euchromatin appeared abundant and uniformly distributed, occasionally with heterochromatin clustered in clumps or located as marginal patches under the nuclear membrane (Figure 10A–C); sometimes dense nucleoli were visible. The junctional complexes between neighbouring epithelial cells appeared well-formed and constituted, as described in the control group, by zonulae occludens followed by zonulae adherens (Figure 10A,C,D). No DISs were observed, no mitophagic vacuoles were found, and only a few vesicles were present.

## 4. Discussion

The present study demonstrates the presence of morphofunctional alterations in mouse PCOS oviducts and the protective action of a formulation of LC-ALC in ameliorating the observed detrimental changes. Based on the evidence of oviductal hyperfibrosis, hyperplasia, altered steroidogenesis, and oxidative and glycative stress, associated with profound morphological alterations, this study confirmed the changes previously observed in the uteri of a DHEA-induced mouse model of PCOS [25].

These conditions together with hypertrophy were also observed in the oviducts of the DHEA group. Hyperfibrosis was associated with an increased thickness of the epithelial tissue that can be responsible for a reduced diameter of the oviductal lumen and permeability, thus compromising the physiology of this reproductive tract, whose contractility and peristalsis is essential for the oocyte/early embryo transport towards the uterine cavity or spermatozoa transport towards the abdominal opening of oviducts or fallopian tubes in women [26,27]. Moreover, we found a reduction in the oviductal cilia in the DHEA group, in agreement with the previous data on the negative effects of testosterone on cilia beating [22].

The morphological alterations here found via light microscopy were associated with the functional data. Since one of the most important characteristics of PCOS is hyperandrogenism [28], steroidogenesis was evaluated via the immunohistochemical and morphometric analysis of 17β-HSD IV, involved in the conversion of androstenedione in DHEA [29]. Its significant upregulation detected in the oviducts of the DHEA group confirms the hormonal imbalance.

The presence of oxidative and glycative damage in the DHEA oviducts was confirmed by the overexpression of (1) the 4-HNE adduct, one of the most important markers for lipid peroxidation, also potentially involved in concomitant diabetes in PCOS women [30] and (2) MG-AGEs, responsible for a positive feedback loop leading to oxidative stress [31].

In agreement with the hypothesis that abnormal mitochondrial metabolic markers, and the related genes, are associated with and participate in the occurrence of PCOS [32], significantly low Tomm20 levels were observed in the DHEA group.

In the present study, we also performed an ultrastructural analysis of the transversal sections of the murine oviducts to have a closer look at the oviductal layers. In the mucosa layer, ciliated epithelial cells showed, in all the groups, the presence of well-preserved mitochondria, nuclei, ER, Golgi apparatuses, multivesicular bodies, and microvilli. However, the TEM analysis revealed a reduced density of the ciliary coverage and a higher presence of mitophagic vacuoles in the DHEA group. Both are signs of the inefficient transport of germ cells and unbalanced energetic support for them [33,34]. Moreover, the DHEA group showed the abnormal presence of DISs between neighbouring epithelial cells. This phenomenon may be responsible for the thickening of the mucosa layer observed in the PCOS model, and it was not observed in the controls and carnitine-treated mice. The junctional complexes are responsible for the tight cellular adhesion, providing a physical barrier. A DIS is a hallmark of damaged junctional complexes, inducing an altered paracellular permeability and limiting the tissue’s ability to directly resist injury [35].

Carnitine supplementation has been successfully employed in PCOS patients to ameliorate the altered hormonal and metabolic parameters, as well as for lipid and bodyweight reduction [36]. Here we found that carnitine treatment can reduce hyperfibrosis, hyperplasia, and hypertrophy and increase the collagen deposition as well as oxidative and glycative stress in the PCOS oviducts. This was also confirmed by the TEM observations of the tunica mucosa, which appeared well-preserved with numerous cilia as in the controls with no evident signs of ultrastructural alterations.

It should also be taken into account that hyperplasia may evolve into atypical forms progressing to in situ carcinoma to potentially become invasive [37]. Along with the ovarian surface epithelium, the oviductal tissue is considered the main candidate for the origin of high-grade serous ovarian cancer. Nevertheless, the relationship between PCOS and ovarian cancer is still under debate. We demonstrated that the administration of both LC and ALC ameliorated hyperfibrosis, hyperplasia, and altered steroidogenesis; carnitines also improved the mitochondrial metabolism, and reduced the oxidative and glycative stress. Furthermore, the TEM analysis revealed a better mucosa layer with closer cellular adhesions.

## 5. Conclusions

Our results evidence that the murine oviducts from the DHEA-induced model of PCOS are affected by hyperfibrosis, hyperplasia, and hypertrophy. Oxidative and glycative alterations were associated with MG-AGE accumulation and lipid peroxidation. The oral supplementation of carnitines reduced the detrimental effects found in the DHEA group. The TEM analysis confirmed that carnitine supplementation restored an ultrastructural aspect of ciliated oviductal cells to one more comparable to the controls, as also indicated by the well-preserved mitochondria, numerous cell junctions, and a continuous ciliar coverage in the DHEA/LC-ALC group. Moreover, mitophagic vacuoles were reduced, and dilated intercellular spaces were absent.

In conclusion, the present research supports that carnitines as a dietary supplement may be beneficial in reducing some of the morphofunctional detrimental effects connected to PCOS in the reproductive system. Considering that the ampullar lumen is the site where spermatozoa fertilize the oocyte and that the subsequent transport of the embryo in the uterus is driven by the oviducts, these findings may be useful for an alternative treatment protocol in PCOS-connected female infertility.

## Figures and Tables

**Table 1:** Morphometric analysis of the epithelium, tunica mucosa, and ampullar wall. Values are expressed as mean ± SD. Differences were evaluated via ANOVA followed by Tukey’s HSD post-hoc test. Superscripts indicate a significant difference in the same row (*p* < 0.05).

	Control	DHEA	DHEA/LC-ALC
EPITHELIUM	19.132 ± 5.982 ^a^	36.971 ± 5.565 ^b^	22.585 ± 7.814 ^a,b^
MUCOSA	28.943 ± 5.323 ^a^	42.323 ± 1.535 ^b^	34.399 ± 6.628 ^a,b^
AMPULLARY WALL	63.771 ± 8.608 ^a^	86.393 ± 11.209 ^b^	73.117 ± 8.949 ^a,b^

**Table 2:** Morphometric analysis of the immunohistochemical markers assessed on transverse sections of ampullar oviducts. Values are expressed as mean ± SD of mean pixel intensity. Differences were evaluated via ANOVA followed by Tukey’s HSD post-hoc test. Superscripts indicate a significant difference in the same row (*p* < 0.05).

	Control	DHEA	DHEA/LC-ALC
Col-1	114.198 ± 14.431 ^a^	151.102 ± 6.069 ^b^	134.757 ± 12.856 ^a,b^
17 β-HSD IV	112.175 ± 18.9 ^a^	159.320 ± 10.313 ^b^	140.502 ± 16.471 ^b^
4-HNE	87.178 ± 6.318 ^a^	151.149 ± 1.590 ^b^	148.670 ± 5.831 ^a,b^
TOMM 20	163.698 ± 2.331 ^a^	128.366 ± 3.474 ^b^	155.314 ± 9.732 ^a^
MG-AGE	126.053 ± 12.862 ^a^	140.427 ± 5.868 ^b^	133.279 ± 15.217 ^a,b^

## Data Availability

Data supporting the findings of this study are available from the corresponding author upon reasonable request.
